# Microstructural Characterization of Alkali-Activated Composites of Lightweight Aggregates (LWAs) Embedded in Alkali-Activated Foam (AAF) Matrices

**DOI:** 10.3390/polym14091729

**Published:** 2022-04-23

**Authors:** Katja Traven, Wolfgang Wisniewski, Mark Češnovar, Vilma Ducman

**Affiliations:** 1 Slovenian National Building and Civil Engineering Institute (ZAG), Dimičeva 12, 1000 Ljubljana, Slovenia; info@zag.si (K.T.); wolfgang.wisniewski@zag.si (W.W.); mark.cesnovar@zag.si (M.Č.); 2 International Postgraduate School Jožef Stefan, Jamova 39, 1000 Ljubljana, Slovenia

**Keywords:** alkali-activated foam (AAF), lightweight aggregate (LWA), mechanical properties, thermal insulation, LWA–AAF interface

## Abstract

Alkali-activated composites of lightweight aggregates (LWAs, with beneficial insulating properties) and alkali-activated foams (AAFs, higher added value products due to their production from waste materials at well below 100 °C) allow for the expectation of superior properties if a chemical bonding reaction or mechanical interlocking occurs during production. However, the interfaces between LWAs and an AAF have not been studied in detail so far. Chemical reactions are possible if the LWA contains an amorphous phase which can react with the alkaline activators of the AAF, increase the bonding, and thus, also their mechanical strengths. These, in turn, allow for an improvement of the thermal insulation properties as they enable a further density reduction by incorporating low density aggregates. This work features a first-detailed analyses of the interfaces between the LWAs’ expanded polystyrene, perlite, expanded clay and expanded glass, and the alkali-activated foam matrices produced using industrial slags and fly ash. Some are additionally reinforced by fibers. The goal of these materials is to replace cement by alkali-activated waste as it significantly lowers the environmental impact of the produced building components.

## 1. Introduction

Because the building sector has been recognized to be one of the major contributors to global warming, finding alternatives to conventional building materials is receiving increasing attention. Alkali-activated materials (AAMs) present promising substitute materials as their lower energy demand during production causes a smaller CO_2_ footprint. In the most general description, AAMs are inorganic systems consisting of two main components: a reactive solid precursor such as metakaolin, slags or ashes, and an alkaline activator solution such as Na_2_SiO_3_, K_2_SiO_3_, NaOH, or KOH [1]. Adding a foaming agent to this basic mixture leads to materials denoted as alkali-activated foams (AAFs) [2,3]. AAFs represent higher added value products due to their low production temperature of well below 100 °C, but still show properties comparable to foamed glasses or ceramics which are produced at above 900 °C.

AAFs can find various applications as catalysts, adsorbents, bone scaffold materials, filtration membranes, or thermal/acoustic insulators [2,4] and can be produced by different routes [2]. Of these, direct foaming is most commonly applied; here foaming agents such as Al, SiC, Si, NaOCl, FeSi alloys, NaBO_3_, or H_2_O_2_ are added to the alkali-activated slurry to trigger a chemical reaction which releases gaseous products. The gasses are trapped in the material’s structure during hardening which results in a highly porous material [5,6]. Surfactants or stabilizing agents such as sodium oleate, sodium dodecyl sulfate, triton, or stearic acid are added to the slurry to stabilize the pores and control their size [7,8]. However, several studies have shown that the compressive strength of AAFs, usually ranging from 1 MPa–10 MPa with densities of 360–1400 kg/m^3^, decreases with a density reduction independent of the production method or used additives [9,10]. Lightweight aggregates (LWAs) are construction materials with a reduced bulk density, and their key physical properties are their bulk density, specific gravity, unit weight, porosity, and water absorption [11]. They are widely applied for geotechnical fills, insulation products, soil engineering, hydroculture, drainage, roof gardens, or filters in several industries [12]. LWAs can either be produced using natural rock by crushing and sieving scoria, pumice, breccias, tuff, or volcanic cinders or by thermally treating naturally occurring materials (e.g., vermiculite, clay, perlite, shale, slate) or industrial by-products (e.g., fly ash, blast furnace slag, industrial waste, sludge) [11]. Artificial LWAs can be manufactured by either expansion or agglomeration. Expansion, as in expanded glass, occurs when a material is heated to a fusion temperature where it becomes pyro-plastic with a simultaneous formation of gas, released from added or intrinsic foaming agents. During agglomeration, the powdered material is bound together by either sintering mechanisms or cold bonding processes including additive binders [13,14,15,16]. Currently, the most used and valued manufactured LWAs from natural source materials are shale and expanded clay. However, LWAs can also be produced by applying the alkali-activation process to industrial waste such as fly ash, ground granulated blast slag, or rice husk ash [17]. A polymer LWA extensively used in building and construction is expanded polystyrene (EPS). Its manufacturing process begins with small polystyrene beads ca. 200 µm in diameter which are permeated with a foaming agent, most commonly pentane, and expanded using steam [15]. EPS is widely used in construction for external thermal insulation panels due to its energy efficiency, but it also finds application as the aggregate in lightweight concrete, decorative tiles and molding, panels, and embankment backfilling [16,18].

Several LWAs, such as recycled lightweight blocks [19], Petrit T [20], pumice aggregates [21], vermiculite [22], cork [23], Etna volcanic aggregates [24], and water reservoir sediments [25] have been combined with AAMs. Optimized foamed thermal insulation materials produced by the alkali-activation process using Na_2_SiO_3_ and unexpanded ground waste, perlite, and rock wool showed a low thermal conductivity of 0.040–0.060 W/mK, a low density of 0.1–0.2 g/cm^3^, and compressive strengths from 0.09 to 0.60 MPa [26]. Foamy alkali-activated materials have been produced from nonexpanded perlite and show a thermal conductivity of 0.030 W/mK, a compressive strength of 0.78 MPa, and superior fire resistant properties, i.e., they are 100% noncombustible and categorized as the fire class A1 [27]. A similar material (density 0.46 g/cm^3^, thermal conductivity 0.084 W/mK, compressive strength 1.6 MPa) was produced using expanded perlite and K_2_SiO_3_ as the activator [28].

Environmentally friendly, lightweight foamed geopolymer composites have also been produced as a thermal insulating material using H_2_O_2_ as the foaming agent, fly ash and metakaolin as precursors, and expanded polystyrene as LWAs [29]. They showed densities of 0.30 to 0.65 g/cm^3^, compressive strengths of 2.0 to 5.5 MPa, and thermal conductivities of 0.122 to 0.195 W/mK. [30]. Exposing fly ash-based geopolymer concretes containing quartz aggregates or expanded clay to temperatures of up to 750 °C showed that the dehydration of capillary water caused cracking accompanied by a loss of strength below 300 °C whereas temperatures above 500 °C caused a sintering-promoted strength increase [31]. Monolithic geopolymer-expanded glass composites have been prepared for the methylene blue removal from wastewaters [32]. Here, adding expanded glass positively affected the removal efficiency.

Although composites of LWAs and cement are being applied on an industrial scale, composites of LWAs and AAMs are still under development. Adding LWAs to cements has been shown to counteract shrinkage [33] and comparable benefits are to be expected in LWA–AAF composites. Replacing cements by AAMs and using waste materials as LWAs significantly lowers the environmental impact of these materials. Superior properties can be expected if a chemical bonding reaction or mechanical interlocking occurs between their components. This should increase their relative mechanical strength, allowing lower densities and with that, enhanced thermal isolation and lighter building components.

The interface between AAMs and aggregates has barely been analyzed and the literature presenting such interfaces usually features dense AAMs and relatively dense aggregates as their interfacial transition zone (ITZ) is easier to analyze. Just as cements, AAMs can form a chemically and structurally modified ITZ to aggregates they are in contact with. The ITZ in an AAM was found to be comparably dense, free of unreacted binder grains due to the “wall effect” and composed of a Na_2_O–CaO–Al_2_O_3_–SiO_2_–H_2_O (N–C–A–S–H) gel [34]. A gradual enrichment of Si and Na has been measured at the interface to quartz sand aggregates spanning 20–50 µm [34]. Low Ca alkali-activated cements where the raw materials contained less than 4 wt% Ca did not form a discernible Ca-enriched ITZ [35]. Another alkali-activated cement did contain high levels of Ca, but an enrichment at the ITZ was not detected [36].

The work presented here is aimed at developing and characterizing LWA–AAM composite materials competitive to some commercially available products. They are energetically advantageous as they are manufactured below 100 °C and based on waste materials instead of cement. The LWAs expanded glass (EG), expanded clay (EC), expanded polystyrene (EPS), and expanded perlite (P) are included to reduce their overall densities and increase insulation while ensuring a sufficient mechanical strength. The performed analyses provide a first insight of the detailed microstructure at the interface between the well-known LWAs and an AAF. Furthermore, possible chemical interactions are analyzed and discussed.

## 2. Materials and Methods

Electric arc furnace slag (slag A), ladle slag (slag R), and fly ash (FA) were used as raw materials. The slags were received as aggregates from Slovenian metallurgical steel and iron plants and milled into powders with a grain size of less than 63 µm. Previously characterized FA from a Slovenian thermal power plant containing akermanite-gehlenite, quartz, anhydrite, hematite, magnesioferrite, and mullite [37] was also used. It contained more than 70 ma% of an amorphous phase suitable for alkali activation [37]. These raw materials were weighed using scale 1 (XPE205, Mettler-Toledo, Trzin, Slovenia, ±0.0001 g), heated to 950 °C for 1 h in a 25 mL Pt crucible and then weighed again to determine their loss on ignition (LOI) components, which amounted to 14.15 ma% for slag A, 20.47 ma% for slag R, and 0.51 ma% for the FA.

The precursors “slag A-p”, “slag R-p” and “FA-p” were produced in batches containing 0.946(9) g of the respective raw material and 9.469(0) g of the flux agent FX-X50-2 (i.e., 50% Li-tetraborate and 50% Li-metaborate, Fluxana GmbH & Co. KG, Bedburg-Hace, Germany) weighed using scale 1. Some of the mixture was placed in a 25 mL Pt crucible and heated to 1100 °C in an XRF xrFuse1 electric furnace (Thermo Fisher Scientific Inc., Ecublens, Switzerland), where it was held for 5 min and shaken for another 8 min before the furnace was turned off, allowing the batches to cool. Then, the chemical compositions of the raw materials were determined using a ARL PERFORM’X sequential X-ray fluorescence (XRF) Spectrometer (Thermo Fisher Scientific Inc., Ecublens, Switzerland) using the UniQuant 5.00 software (Thermo Fisher Scientific Inc., Walthem, MA, USA).

**Figure 1:**
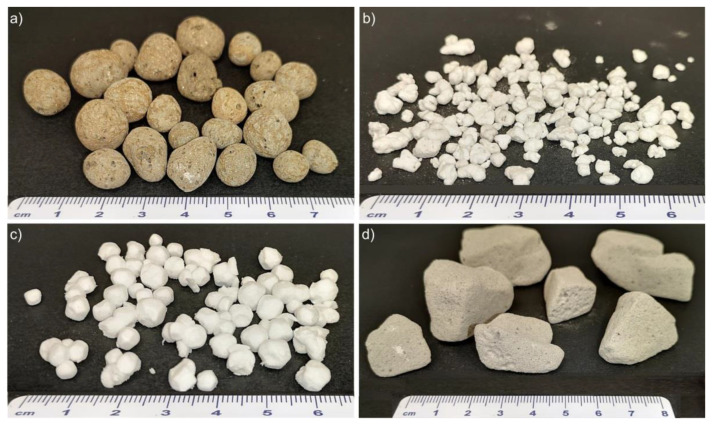
Photographs of the applied LWAs: (**a**) expanded clay, (**b**) perlite, (**c**) expanded polystyrene and (**d**) expanded glass.

The preparation of these composites is also described in the Slovenian patent No. SI 26042 (A) [38]. Dry mixtures of slag powders (grain size < 90 µm) with the optimized slag A-p/slag R-p = 1/1 ratio reported in Ref. [39], FA-p and sometimes polypropylene fibers (Belmix, Mouscron, Belgium) with an average length of 11 mm and a density of 0.94 g/cm^3^ were added. These were mixed with sodium water glass Crystal 0112 (Na_2_SiO_2_ containing 30.4 ma% SiO_2_, 15.4 ma% Na_2_O, and 54.2 ma% H_2_O, Tennants distribution, Ltd., Manchester, UK) and solid NaOH (Donau Chemie, Vienna, Austria) before stirring the batch to homogenize it as well as possible. Then the foaming agent, solid sodium perborate (Belinka Perkemija, Dol, Slovenia), or liquid H_2_O_2_ (Belinka Perkemija, Dol, Slovenia), and the stabilizing agent liquid Triton™ X-100 (Merck, Darmstadt, Germany) were added. Finally, the LWAs expanded clay (Glinopor Vetisa d.o.o., Zalec, Slovenia), perlite (Njiva d.o.o., Zalec, Slovenia), expanded polystyrene (JUB, Dol, Slovenia) or expanded glass (Glasopor AS, Oslo, Norway) presented in Figure 1 was mixed into each batch.

Samples were produced by casting these mixtures into silicone molds and drying them for three days in a WTB laboratory dryer chamber (Binder, Tuttlingen, Germany) at 70 °C and ambient humidity. The components were weighed using scale 2 (Exacta 2200 EB, Tehtnica, Trzin, Slovenia, ±0.01 g) and combined to produce each sample according to Table 1.

The flexural and compressive strength were determined using a Toninorm press (Toni Technik, Berlin, Germany, force detection limit 100 N) with a force application rate of 0.05 kN/s by the standard method [40] and averaged from four test specimens of 20 × 20 × 80 mm^3^. Geometrical densities were determined by weighing individual samples (size of 20 × 20 × 80 mm^3^) and dividing their weight by their volume. Sample dimensions were measured using a Vernier Calliper (Mitutoyo, Neuss, Germany) with a precision of ±0.01 mm. Thermal conductivities were measured using a HFM 446 (Lambda Small, Stirolab, Sezana d.o.o., Slovenia, ±1%), according to EN 12667 and ASTM C518 in ISO 8301.

Optical microscopy of the material cross-sections was performed using a SMZ25/SMZ18 stereo microscope (Nikon, Minato, Japan) at a working distance of 60 mm, images were captured using a digital MikroCamII Microscope Camera (Leica, Wetzlar, Germany). Cross-sections of selected samples were cut and embedded in EpoThin resin (Buehler, Leinfelden-Echterdingen, Germany), cured at 50 °C, and polished using decreasing grain sizes to a final step of ca. 10 min on a SiC Buehler Micro Cut plate 30-10-4000 (Buehler, Leinfelden-Echterdingen, Germany, ca. 5 µm grain size). Scanning electron microscopy (SEM) was performed using a JSM-IT500 (Jeol, Tokyo, Japan) in low vacuum mode. Energy dispersive X-ray spectroscopy (EDXS) was performed using an Ultim Max 65 detector (Oxford Instruments, Abingdon, UK) and the software Aztec 5.0 (Oxford Instruments, Abingdon, UK). SEM figures and EDXS maps were acquired using an acceleration voltage of 15 kV whereas EDXS spot measurements were performed using 10 kV to reduce the information volume.

## 3. Results and Discussion

The compositions of the prepared precursors were determined using XRF and are stated in Table 2.

**Figure 2:**
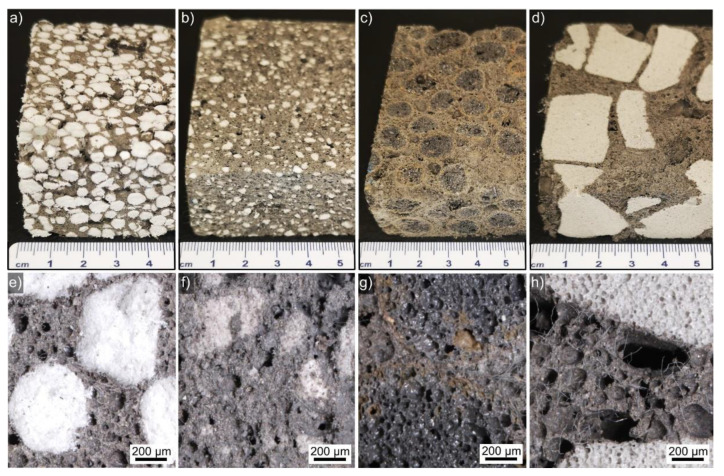
Photographs of LWA–AAF composites containing (**a**) EPS, (**b**) perlite, (**c**) EC, and (**d**) EG. Optical micrographs of cross sections prepared from the composites containing (**e**) EPS, (**f**) perlite, (**g**) EC, and (**h**) EG reinforced by fibers are presented below.

Photographs of selected basic LWA–AAF composites are presented in Figure 2 containing the LWAs (a) expanded polystyrene, (b) perlite, (c) expanded clay, and (d) expanded glass. They are distributed relatively homogeneous in the AAF matrix but the larger LWA size in Figure 2d also leads to larger areas only filled with AAF. Optical micrographs of cross sections prepared through comparable composites reinforced by fibers are presented in the Figure 2e–h; they imply a good adhesion between all LWAs and their AAF matrix. It seems the large LWA size note above also allows for the formation of large pores such as those in Figure 2h exceeding ca. 200 µm in diameter.

### 3.1. Mechanical Properties

The flexural (σ_FS_) and compressive (σ_CS_) strengths listed in Table 3 were measured for the respective materials and the respective standard deviations are stated. The composites containing expanded clay or expanded glass showed higher flexural and compressive strengths than the composites containing smaller LWAs. Although the flexural strength of the fiber-free sample EG1 is below the detection limit and adding fibers would seem to improve this, the sample EC4 also shows a flexural strength below the detection limit although it contains fibers. Perhaps the lower performance of the samples EC4, P3, and EG1 was caused by some random weakness (e.g., a crack) in the samples rather than their microstructure. The measured compressive strengths of the composites were lower than those reported for the respective LWAs [33] except for expanded glass containing samples EG2 and EG3. Assuming the applied LWAs are comparable, this indicates that the matrix can have a stabilizing effect if the LWA is especially brittle.

### 3.2. SEM Analysis

**Figure 3:**
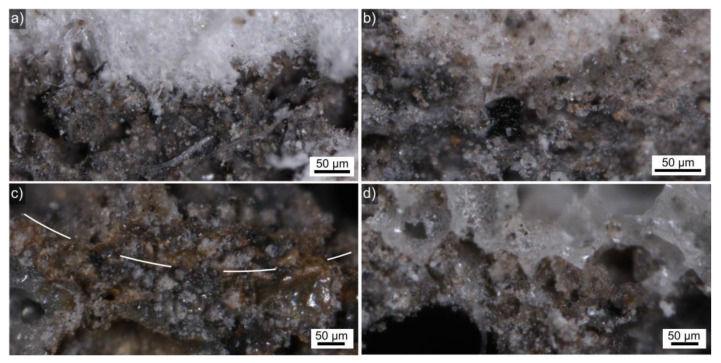
Optical micrographs of selected fiber-reinforced LWA–AAF composite cross sections containing the LWAs (**a**) expanded polystyrene, (**b**) perlite, (**c**) expanded clay, and (**d**) expanded glass. The LWA is always at the top of the image and the dashed line in (**c**) is inserted as a guide for the eye, outlining the circular boundary of an expanded clay particle.

Figure 3 presents optical micrographs visualizing the LWA–AAF interfaces in cross sections through prepared composites reinforced with fibers. There were no discernible shrinkage gaps between the LWAs and their matrix.

**Figure 4:**
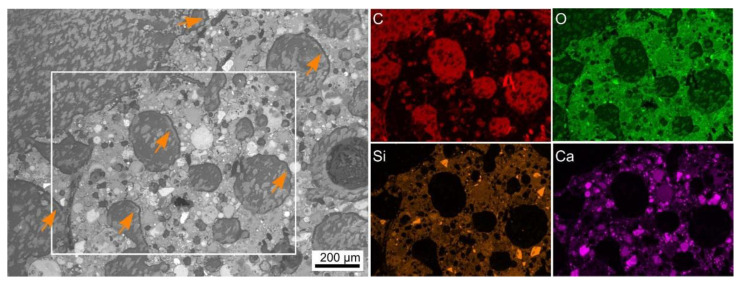
SEM micrograph featuring a cross section though the expanded polystyrene-AAF composite presented in Figure 2a. Arrows highlight a gap between the LWA and its matrix. The framed area was scanned by EDXS and element maps of C, O, Si, and Ca are presented.

SEM analyses were applied to gain further insight into selected LWA–AAF interfaces. Figure 4 presents results obtained from the polystyrene–AAF composite after embedding. The C-map of the scanned area enables to conclude that the spherical domains in the SEM micrograph represent the expanded polystyrene, probably saturated with the embedding polymer, which also contains areas of elevated oxygen content. The AAF matrix shows a comparably complex microstructure in the SEM micrograph, but contains a recognizable distribution of Si and Ca with particles enriched in the respective elements distributed throughout its microstructure. The Ca-rich particles can be assumed to represent unreacted slag [34]. A discernible gap between the polystyrene and the AAF is highlighted by arrows, which could indicate that these components neither formed a chemical bond nor a mechanical interlock during production. However, a comparable gap is not discernible in Figure 3a, so it is possible that these gaps are an artifact introduced by preparing the sample for analysis in the SEM.

**Figure 5:**
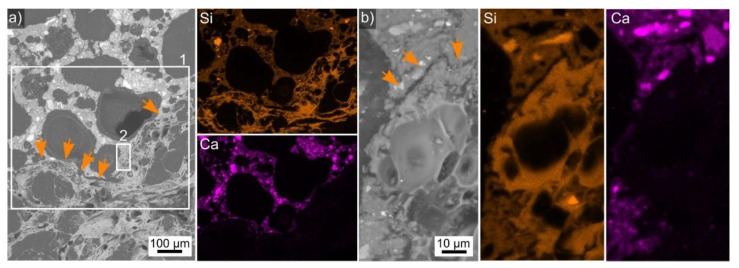
(**a**) SEM micrograph featuring a cross section though the perlite-AAF composite presented in Figure 2b. The area in frame 1 was scanned by EDXS and element maps of Si and Ca are presented. Arrows highlight the boundary between perlite and the AAF. (**b**) The area in frame 2 presented in greater detail along with EDXS element maps of Si and Ca of this area.

An overview of the perlite-AAF composite microstructure is presented in Figure 5a. The large, dark structures in the SEM micrograph are pores filled with the embedding polymer. The boundary between perlite and the AAF is marked by arrows as it is not trivial to discern. However, the presented element maps show that perlite contains more Si than the AAF and no Ca. This interface is so irregular due to the huge pores in both the LWA and the AAF that a ITZ analysis comparable to denser materials [34,35,36] is not feasible. It also shows wide gaps, but there are areas where the components seem to be in direct contact. One such area is highlighted by frame 2 and presented in greater detail in Figure 5b. The SEM micrograph shows a clearly discernible gap between the AAF (top) and perlite (middle).

**Figure 6:**
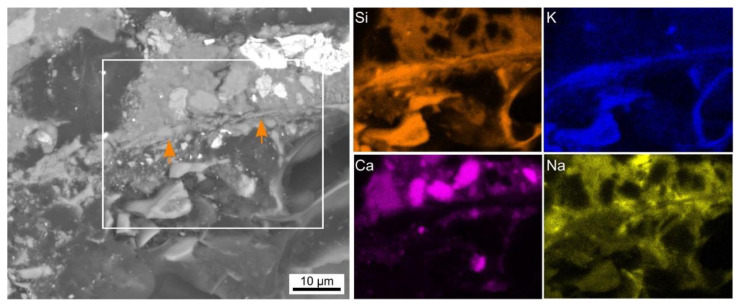
SEM micrograph containing a feature parallel to the perlite (**bottom**)—AAF (**top**) interface and highlighted by arrows. The framed area was scanned by EDXS and element maps of Si, K, Ca, and Na are presented.

On the other hand, this microstructure also contained interface domains similar to those presented in Figure 6, where an apparently compact feature forms this interface in the SEM micrograph. The presented element maps indicate that this feature contains higher amounts of Si and K whereas Ca and Na occur in smaller amounts. As perlite is not prone to shape changes under the given conditions, the Ca-rich domain at the interface could have resulted from a chemical reaction between filler and matrix. EDXS spot measurements were performed along three such interfaces and the resulting composition is stated in Table 4. The compositions are comparable considering the margin of error (assuming to range from ±1–2wt% given an accuracy of 2–5% for the standardless quantification, an unknown sample homogeneity and data acquisition under low vacuum conditions).

**Figure 7:**
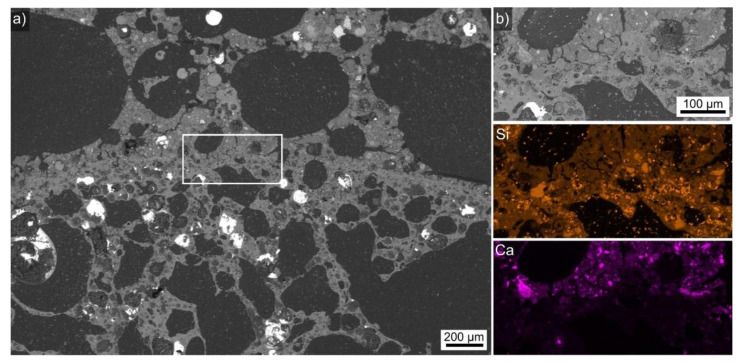
(**a**) SEM micrograph featuring a cross section through the expanded clay-AAF composite presented in Figure 2c. (**b**) The framed area in in greater detail. EDXS element maps of Si and Ca are presented.

An overview of the expanded clay-AAF composite microstructure is presented in Figure 7a. As noted above, the comparably large LWA particle size allowed for the formation of huge pores in the AAF. A slightly rounded, rather compact interface was discernible between the AAF and the LWA, also containing very large pores. The framed area is presented in greater detail in Figure 7b and the element maps of this area presented below show that the expanded clay contains Si-enriched particles, but otherwise produces a very similar Si signal. The element map of Ca allowed for a clear identification as the Ca-enriched particles characteristic of the AAF only occurred on top of this interface, identifying this microstructure as the AAF.

**Figure 8:**
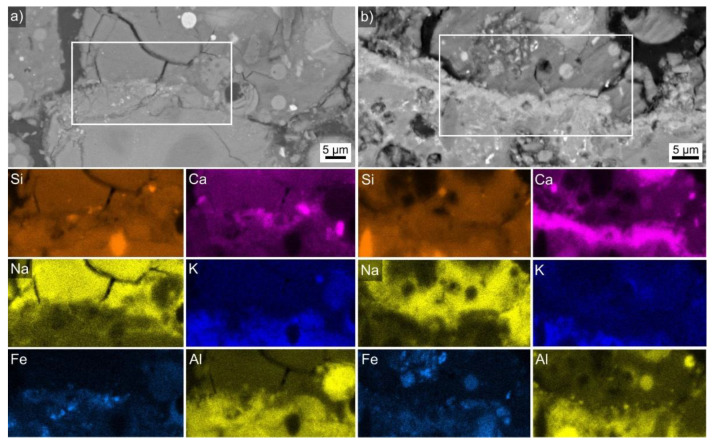
(**a**) SEM micrograph of the AAF (**top**)—expanded clay (**bottom**) interface, element maps of an EDXS scan performed on the framed area are presented below. (**b**) The same, but at a different location.

Two detailed SEM and EDXS analyses of the expanded clay-AAF interface are presented in Figure 8a,b. Although the SEM micrograph clearly shows that the AAF was in direct contact with the expanded clay, only the element map of Ca shows a systematically enhanced signal along this interface, which could indicate a chemical reaction between filler and matrix in this composite. In combination with the interface morphology, the detected Ca enrichment implies that a Ca-enriched zone comparable to the gel noted in Ref. [34] was formed. EDXS spot measurements were performed along three such interfaces and the resulting composition is stated in Table 5. Noteworthy differences are an elevated amount of Si in Figure 8a, and clear variations in the measured Ca content.

**Figure 9:**
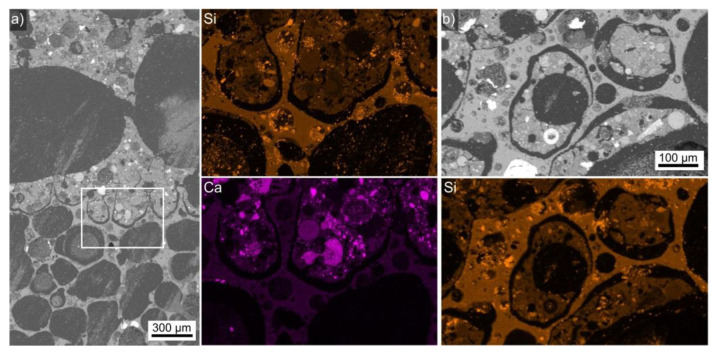
(**a**) SEM micrograph featuring a cross section through the expanded glass-AAF composite presented in Figure 2d. The framed area was scanned by EDXS and element maps of Si and Ca are presented. (**b**) SEM micrograph of a segment where the AAF is completely surrounded by the expanded glass in the prepared cross section. The Si element map allows for the component attribution.

An overview of the expanded glass–AAF composite microstructure is presented in Figure 9a; the AAF amongst these very large LWA particles contains the largest pores of all presented materials. Element maps of Si and Ca acquired in the framed area are presented, and they show that the expanded glass (bottom) contains more Si but less Ca than the AAF (top). It is also very clear that these components are usually separated by a rather wide gap. However, the AAF expanded into the pores of the expanded glass very well, mechanically interlocking their microstructures. This is illustrated by Figure 9b, which features two AAF segments completely surrounded by expanded glass in the prepared cross section, as visualized by the presented element map of Si.

### 3.3. Thermal Conductivity

The thermal conductivity of materials is affected by a wide range of parameters ranging from the atomistic scale, i.e., the dominant atomic bonding type, over the µm-scale, via the degree of crystallinity, to macroscopic parameters such as the porosity and its type as well as the overall density. If, for example, materials of a different “nature” such as “clay brick”, a “Ca-silicate unit”, or a “densely aggregated concrete” all show matching densities of 1600 kg/m^3^, then their respective thermal conductivities amount to 410, 550, or 690 mW/mK according to the tables A.1, A.2, and A.3 in EN 1745 [41].

Thermal conductivities for the produced materials were assessed based on EN 1745 [41], which contains tables with generic values for masonry materials in dependence on their density. If the measured densities did not match those in the selected tables A.4, A.5, or A.6 of EN 1745 [41], the values of the most comparable materials were linearly extrapolated based on the density. The thermal conductivities of the samples EC1–EC4 were measured for comparison; the maximum difference to the assessed values is 27 mW/mK for sample EC3. The respective sample dimensions, weight, calculated density, and thermal conductivity are stated in Table 6. The density of the produced materials is generally somewhat higher than comparable materials prepared using cement [33].

**Figure 10:**
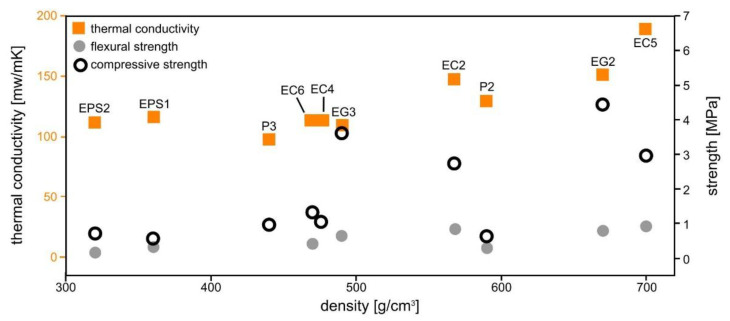
Thermal conductivity and flexural as well as compressive strength correlated to the density of selected samples.

Figure 10 presents the assessed thermal conductivity and measured strengths of selected materials correlated to their density, and confirms the expected correlation between them: a lower density is accompanied by lower strengths and usually also a lower thermal conduction, although it must be noted that samples P3, EG3, and EG6 have lower values than EPS1, which has a much lower density. Here the “nature” of the materials comes into play as EPS1 and 2 contain organic polymers whereas all other samples only contain inorganic-nonmetallic compounds which should show a much better fire resistance than polystyrene. The latter should simply burn in case of a fire, and the formed CO_2_ gas could reduce the mechanical strength significantly by producing internal cracks.

In summary, large LWA particle sizes allow for the formation of larger pores in the AAF, probably because of larger gaps. If the strength of the AAF defined the mechanical properties of the produced composites, such large pores should decrease the overall performance of the material, and adding fibers could be expected to compensate such weak links in the system. Although the optical micrographs in Figure 3 imply a good adhesion between all LWAs and their AAF matrix, the detailed SEM analysis reveals that gaps less than 50 µm wide usually occur between these components. Such small gaps should be difficult to discern in optical micrographs and also occur between cement and polystyrene [42,43], but could also form during sample preparation for SEM analysis. Mechanical interlocking of the AAF with the LWA of course depends on the LWA structure, but the large pores in the expanded glass allow for the AAF to expand into them, causing the obvious interlock discernible in Figure 9.

Direct contacts between LWA and AAF were found in the composites containing perlite or expanded clay. In the case of perlite, the features morphologically most likely to have formed at the interface contained elevated levels of Si and K whereas Ca and Na occur less often. The low Ca content in these features caused strong variations in the calculated Ca/Si ratio and indicate that these interfaces are perhaps more comparable to those featured in Ref. [36] where a Ca enrichment was not detected despite high Ca concentrations in the alkali-activated cement.

The mechanical properties of composites containing the large, rather coarse LWAs expanded glass and expanded clay are superior, probably due to a better mechanical interlocking with the AAF matrix. The thermal insulation properties could be improved by a further density reduction, but sufficient compressive (and bending) strengths must be provided to allow for the material to be manipulated during installation and use. Furthermore, other relevant factors such as fire resistance should not be ignored.

## 4. Conclusions

LWA–AAF composites incorporating expanded polystyrene, perlite, expanded clay, or expanded glass were produced. Large LWA particle sizes led to large gaps, which allowed for larger pores in the AAF. Large, open pores in the LWA allowed for the AAF to expand into them, mechanically interlocking the fillers with their matrix. The AAF matrix probably had a stabilizing effect on the brittle LWAs. The huge pores in both the matrix and the filler make comparable analyses of the very inhomogeneous ITZ in these highly porous composites almost impossible. Nevertheless, some chemical interactions were indicated and a Ca enrichment was detected at an expanded clay—AAF interface.

Furthermore, achieving waste-based materials comparable to cement-based composites contributes to the field of environmental protection as well as that of sustainable development in construction.

## Figures and Tables

**Table 1:** Composition of the prepared samples in [g] with H = hydrogen peroxide and PB = sodium perborate. They are denoted according to the applied LWA: EC-expanded clay (EC), perlite (P), expanded polystyrene (EPS) or expanded glass (EG).

Sample	FA-p	Slag Precursor Mix	Na_2_SiO_3_	NaOH	Triton	Foaming Agent/Type	PP-Fibers	LWA
EC1	110.0	/	37.4	4.0	1.5	1.5/H	/	58.0
EC2	110.0	/	37.4	4.0	1.5	1.5/H	0.5	58.0
EC3	/	132.0	72.0	2.0	2.0	4.6/H	/	58.0
EC4	/	132.0	72.0	2.0	2.0	4.6/H	0.5	58.0
EC5	50.0	50.0	54.0	0.6	0.8	0.5/PB	0.5	70.0
EC6	50.0	50.0	54.0	0.6	0.8	2.0/H	0.5	100.0
P1	/	132.0	72.0	2.0	2.0	4.6/H	/	11.8
P2	50.0	50.0	54.0	0.6	0.8	0.7/PB	0.5	30.0
P3	50.0	50.0	54.0	0.6	0.8	2.0/H	0.5	40.0
EPS1	50.0	50.0	55.0	0.6	0.8	1.5/H	0.5	4.0
EPS2	50.0	50.0	55.0	0.6	0.8	1.0/PB	0.5	4.0
EPS3	50.0	50.0	55.0	0.6	0.8	1.0/PB	0.5	4.0
EG1	/	132.0	72.0	2.0	2.0	4.6/H	/	10.0
EG2	50.0	50.0	54.0	0.6	0.8	0.5/PB	0.5	60.0
EG3	50.0	50.0	54.0	0.6	0.8	2.0/H	0.5	80.0

**Table 2:** Chemical composition of the precursors in mass%.

Component	SiO_2_	Al_2_O_3_	Fe_2_O_3_	CaO	MgO	Na_2_O	K_2_O	Cr_2_O_3_	MnO	Others
slag A-p	24.53	9.95	13.25	24.32	17.33	0.15	0.20	4.38	2.61	3.26
slag R-p	17.05	6.48	5.78	34.69	28.96	0.35	0.17	0.22	0.77	5.52
FA	44.83	22.98	10.65	12.38	2.80	1.19	2.20	0.02	0.26	2.68

**Table 3:** Flexural and compressive strengths of selected samples. Standard deviations (std) are stated in brackets.

Sample	σ_FS_ [MPa] (std)	σ_CS_ [MPa] (std)
EC2	0.82 *	2.72 (0.00)
EC4	b.d.l.	1.04 (0.12)
EC5	0.90 *	2.95 (0.26)
EC6	0.39 *	1.31 (0.01)
P2	0.29 *	0.61 (0.05)
P3	b.d.l.	0.95 (0.07)
EPS1	0.34 (0.10)	0.55 (0.09)
EPS2	0.15 (0.01)	0.69 (0.42)
EG1	b.d.l.	0.85 (0.01)
EG2	0.77 *	4.44 (0.28)
EG3	0.62 *	3.62 (1.08)

b.d.l. = below detection limit, * single value or b.d.l.

**Table 4:** Chemical composition determined at three interfaces comparable to that featured in Figure 6a. Only the stated elements were included in the analysis and the values were averaged from at least five spots analyzed at each interface. The average Ca/Si ratio is also stated for each interface.

in wt %	O	Na	Mg	Al	Si	K	Ca	Fe	Si/Ca
interface 1	48	12	1	5	29	2	3	1	10:1
interface 2	50	5	0	6	34	3	2	1	20:1
interface 3	50	5	0	6	33	2	1	1	27:1

**Table 5:** Chemical composition determined at the interfaces featured in Figure 8 and one comparable interface. Only the stated elements were included into the analysis and the values were averaged from at least five spots per interface. The respective average Ca/Si ratio is also stated.

in wt %	O	Na	Mg	Al	Si	K	Ca	Fe	Si/Ca
Figure 9a	48	3	4	7	28	2	5	3	6:1
Figure 9b	45	3	5	6	21	1	14	6	3:2
Comparable interface	44	1	4	6	20	0	20	5	1:1

**Table 6:** Sample dimensions, weight, calculated density, and assessed values of the thermal conductivity of the prepared LWA–AAF composites. Measured values for the thermal conductivity are stated in brackets.

Sample	Width [mm]	Length [mm]	Height [mm]	Weight [g]	Density [kg/m³]	Thermal Conductivity [mW/mK]
EC1	90.80	90.60	28.49	128.87	549.8	140 (134)
EC2	70.50	76.30	21.03	64.23	567.8	148 (140)
EC3	86.60	84.00	35.95	100.45	384.1	96 (123)
EC4	91.80	68.30	27.21	81.20	475.9	114 (130)
EC5	101.95	21.50	20.13	30.79	700	190
EC6	100.50	21.70	29.23	30.19	470	114
P2	102.00	26.00	21.43	33.62	590	129
P3	103.80	21.35	25.77	25.10	440	98
EPS1	99.70	18.23	25.65	17.01	360	116
EPS2	97.44	22.83	20.86	14.88	320	112
EG2	101.95	20.40	21.60	29.93	670	151
EG3	100.25	21.30	22.37	23.38	490	108

## Data Availability

All final data can be provided by authors.
